# Time-of-Flight Detection of Boson Peak in Volcanic Glass

**DOI:** 10.3390/ma19183875

**Published:** 2026-09-11

**Authors:** Yuhei Komaki, Soo Han Oh, Koki Nagao, Tatsuya Mori, Seiji Kojima

**Affiliations:** Department of Materials Science, University of Tsukuba, Tsukuba 305-8573, Ibaraki, Japans2620378@u.tsukuba.ac.jp (K.N.); mori@ims.tsukuba.ac.jp (T.M.)

**Keywords:** time of flight, terahertz spectroscopy, glass, obsidian, boson peak, density of states, light–vibration coupling, dielectric constant

## Abstract

**Highlights:**

• The boson peak of volcanic obsidian glass was observed by the time-of-flight measurement.
• The boson peak frequency was determined to be 1.07 THz, which is lower than that of silica glass.
• The infrared light–vibration coupling coefficient C_IR_ was analyzed by Taraskin’s model.
• The uncorrelated charge fluctuations determined from C_IR_ were much higher than that of silica glass due to a weakening of Si-O bonds in obsidian.

**Abstract:**

A universal excitation called a boson peak has been observed in glasses in the terahertz range. The terahertz time-domain spectroscopy (THz-TDS) enables the time-of-flight (TOF) detection of a boson peak. Volcanic obsidian is a typical natural inorganic glass. The broadband frequency dependence of complex dielectric constants was measured by TOF. The boson peak at 1.07 THz was observed at room temperature, which is lower than 1.2 THz of silica glass. The decrease in BP frequency may be attributed to the modulation of the SiO_4_ network by metal ions in obsidian. However, the IR light–vibration coupling coefficient C_IR_ of obsidian is much higher than that of silica glass. The uncorrelated charge fluctuations, $\sigma_{1}=\sqrt{\langle {q_{1i}}^{2}\rangle }=0.481e$ were determined by C_IR_ in Taraskin’s model. The value of obsidian is much higher than $\sigma_{1}$ = 0.06*e* of silica glass. However, this value is comparable with $\sigma_{1}\approx 0.44e$ of sodosilicate glass, *x*Na_2_O(1−*x*)SiO_2_ at *x* = 0.2. As a common feature in silica-based glasses, the content of metal elements may lead to a nonlocal redistribution of all the atomic charges, which induces a weakening of Si-O bonds, and the uncorrelated charge fluctuations can be enhanced.

## 1. Introduction

Low-energy vibrational excitations in condensed matter have attracted much attention because they give new insights into lattice instability, localized modes, and disordered states. As the universal feature of the low-energy vibrational excitation in non-equilibrium glassy states, the boson peak (BP) in the THz range has been extensively studied by theory [1] and experiments such as Raman scattering, far-infrared spectroscopy, inelastic neutron and X-ray scattering [2,3], and THz time-domain spectroscopy (THz-TDS) [4]. A BP is defined as a peak in g(ν)/ν^2^ deviated from the Debye mode, where ν is a phonon frequency, and g(ν) is the vibrational density of states (VDoS) [1]. A peak in g(ν)/ν^2^ was also observed in the temperature dependence of the heat capacity C_p_ at a constant pressure as a broad peak in C_p_/T^3^ below 10 K. Quasi-localized vibrations that form BP have a characteristic localization length of the order of nanometers, and typically some tens or hundreds of atoms participate in these vibrational modes [5]. The relation between a BP frequency and the first sharp diffraction peak (FSDP) in a static structure factor S(Q), where Q is a wave vector, has been discussed to clarify the origin of BPs in relation to a characteristic localization length [3,6].

Silica (SiO_2_) is the typical glass-forming material, and its glass structures have been extensively studied using X-ray and neutron diffraction measurements. The observed FSDP at Q = 1.51 Å^−^^1^ indicates the intermediate-range order related to a periodicity of 2π/Q = 4 Å (repetitive characteristic distance between structural units). The half-width at half- maximum (HWHM) ΔQ = 0.31 Å^−1^ is related to the coherence length of 2π/ΔQ = 20 Å (structure correlation length of a disordered structure) [7]. The relation between the BP frequency (51.5 cm^−1^) observed by Raman scattering and the medium-range order has been discussed. Silica glass has been extensively used in structural and functional glasses for its broadband transmission and thermal and mechanical stability. As a technologically important glass, silica-based soda–lime–silicate glass is a major manufactured glass and has also been well studied [8].

Natural glasses are aged for many millions of years at temperatures well below a glass transition temperature. They reach thermodynamically quite stable states, which are not accessible under normal experimental conditions. Therefore, natural glasses are important to study the physical properties of glasses. A typical natural organic glass is amber, and a typical inorganic natural glass is obsidian. Amber is a fragile glass, while obsidian is a strong glass, and the physical properties of obsidian are quite different from those of amber. In strong glass, the intensity of a BP is high, while the intensity of the fast relaxation process is low. The intense BP of silica glass has been extensively studied, including the analysis of the infrared (IR) light–vibration coupling coefficient. In contrast, in fragile glass, the BP intensity is very low, while the intensity of the fast process is high [9,10]. The observation of a BP of fragile amber with a fragility index of 90 [11] has occurred only through rapid quenching to suppress the fast relaxation process [12]. However, the intensity of a BP is very weak, and it is difficult to analyze the IR light–vibration coupling coefficient. Obsidian is produced by the rapid cooling of volcanic lava through a glass transition temperature and freezes, not permitting sufficient time for crystal growth [13]. Obsidian is a SiO_2_-based strong glass. Obsidian is primarily composed of SiO_2_ but also contains Al_2_O_3_, K_2_O, Na_2_O, and FeO, etc., and it can be a strong glass in Angell’s classification. Therefore, the BP intensity can be stronger than that of fast relaxation processes, and the observation of an intense BP is expected for the study of the IR light–vibration coupling coefficient.

The recent generation of coherent THz radiation by femtosecond-pulse lasers enables terahertz time-domain spectroscopy (THz-TDS) using the compact photoconductive antennas [14,15]. It enables the time-of-flight (TOF) measurements of elementary excitations such as phonons, plasmons, polaritons, etc. [16,17]. The time-domain signals of the amplitude and phase of coherent THz waves transmitted through or reflected at a sample enable the accurate determination of the real and imaginary parts of the dielectric constant. The frequency dependence of the dielectric constant was determined by Fourier transformation. In research in materials science, THz-TDS has been extensively applied to many kinds of crystals, ceramics, and glasses such as semiconductors [18], superconductors [19], ferroelectrics [20], pharmaceuticals [14,21], and glasses [22].

In the present paper, the temperature dependence of the dielectric properties of volcanic glass obsidian was studied by the TOF measurement using THz-TDS to observe a BP and analyze the IR light–vibration coupling coefficient.

## 2. Experimental Methods

**Figure 1:**
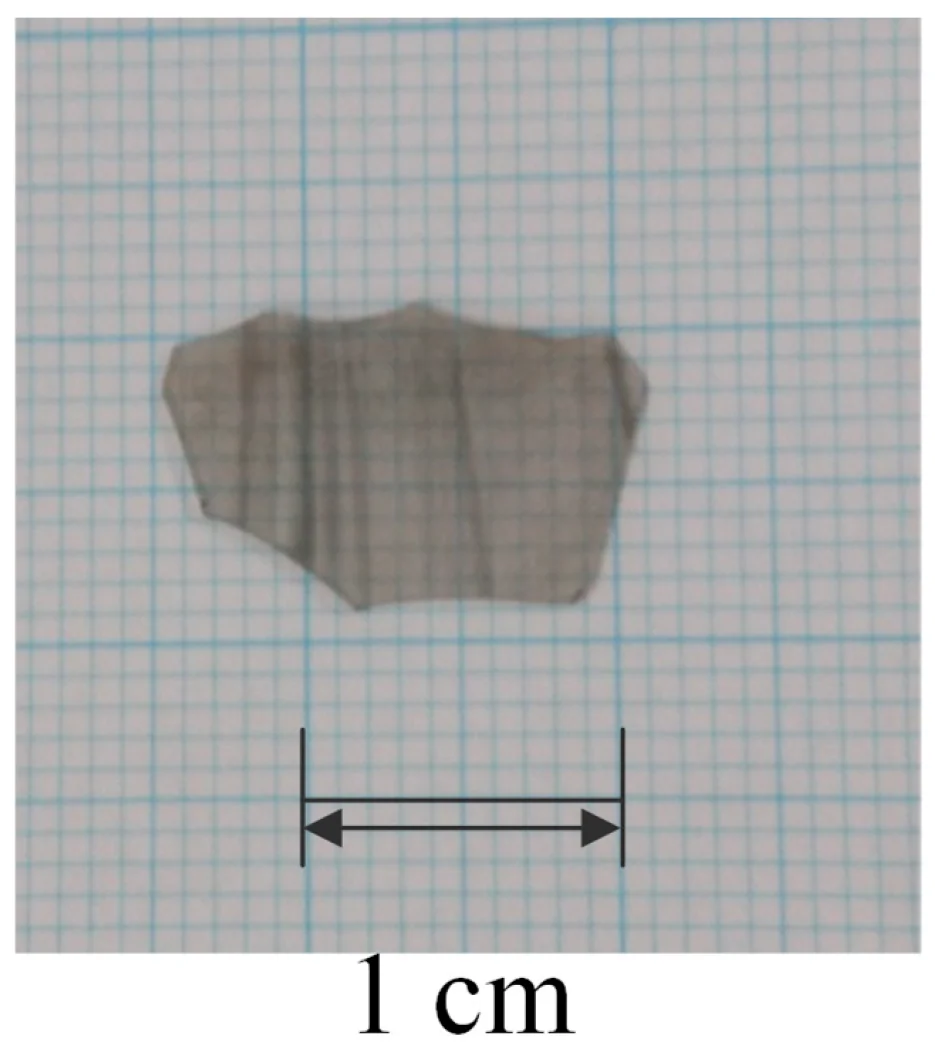
Obsidian plate.

The archaeological obsidian (Figure 1) was obtained from Mexico. The plate used for the THz-TDS measurement and the samples used for the chemical analysis were cut from the same original obsidian block. The bulk chemical composition was determined at the Mie Prefecture Industrial Research Institute by X-ray fluorescence analysis using the glass-bead method. Four subsamples were analyzed, and the average values are listed in Table 1.

The main content is silica glass, described as an infinite random network of SiO_4_ tetrahedra linked together at the corners, while Al_2_O_3_, Na_2_O, K_2_O, etc., are included as the network-modifying cation oxides. The obsidian (Figure 1) was cut and polished for the THz-TDS measurements, and the thickness of the sample was 522 μm, which is appropriate for the good intensity of THz transmission spectra.

**Figure 2:**
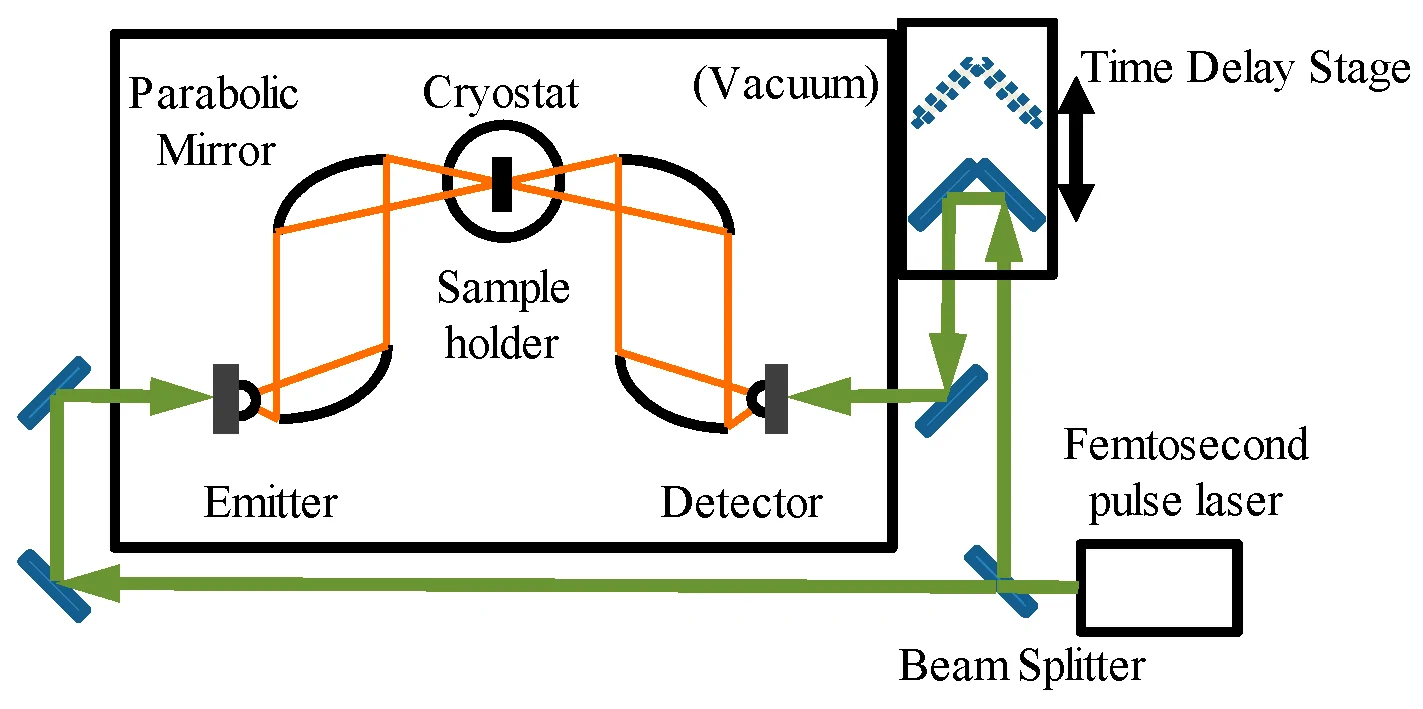
Experimental setup of the THz-TDS spectrometer (RT-10000) and the liquid-He flow cryostat.

A THz-TDS system (RT-10000, Tochigi Nikon Co., Ohtawara, Japan) [4,21] was used with the standard transmission configuration in the frequency range between 0.2 and 4.0 THz as shown in Figure 2. The temperature dependence of the sample was measured using a liquid-He flow cryostat (Helitran LT-3B, Advanced Research Systems, Pelkie, MI, USA) from 27 to 295 K. The low-temperature-grown GaAs photoconductive (PC) antennas were used for the THz pulse emitter and detector. The PC antennas were triggered by a mode-locked Ti: sapphire pulsed laser with a wavelength of 780 nm, a pulse width of less than 100 fs, and a repetition rate of 80 MHz. The THz-wave propagation path was enclosed in a dry air chamber with dry air flow. The broadband spectra between 0.2 and 6.5 THz at room temperature were measured by another THz-TDS system (TAS7500SU, Advantest Corp., Tokyo, Japan) [14]. In the data analysis of the two systems, we smoothly connected the absolute values of the real and imaginary parts of dielectric constants of the same sample at 1.9 THz, using the low-frequency data at room temperature.

## 3. Results

**Figure 3:**
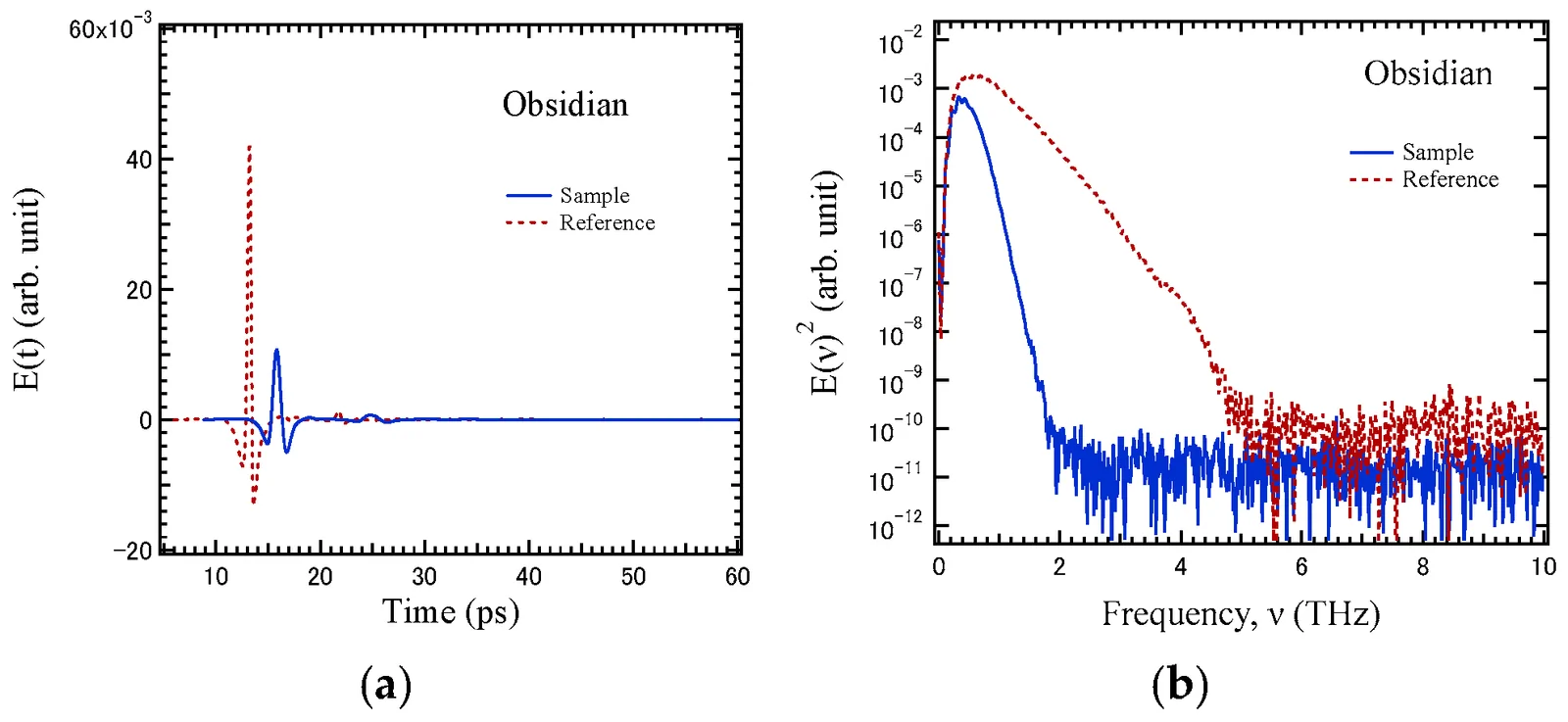
Time-domain and frequency-domain responses of (**a**) *E*(t) and (**b**) *E*(ν)^2^ of obsidian at room temperature by the transmission THz-TDS measurement.

The transmitted time-domain THz *E*-field waveforms through the vacuum are shown as “Reference”, and the transmitted waveforms through the obsidian sample with a thickness of 522 μm are shown as “Sample” in Figure 3a. The *E*-field (reference) shows the delay time caused by the propagation of a THz wave through vacuum. In contrast, the *E*-field (sample) shows the delay time caused by the timeofflight of the propagation of polar phonons in a sample. The timeofflight of phonons in a sample is obtained by the difference in delay times between two transmitted THz waves. The phase velocity of a sample can be measured by the thickness of a sample and the TOF. In order to determine the frequency dependence of phase velocity related to the phonon dispersion relation, the *E*-field waveforms are transferred into the power spectra by the Fourier transformation. Figure 3b shows the frequency–domain power spectra of the sample and the reference.

The time delay of THz transmission $\tilde{t}\left(\nu \right)$ was calculated by the observed time-domain waveforms shown in Figure 3a,b using the following equation,

(1) $$\tilde{t}\left(\nu \right)=\frac{{\tilde{E}}_{sam}\left(\nu \right)}{{\tilde{E}}_{ref}(\nu )}=\frac{4\tilde{n}}{{\left(\tilde{n}+1\right)}^{2}}\exp\left\{i\frac{2\pi \nu }{c}\left(\tilde{n}-1\right)d\right\},$$

where *E*_sam_(ν) and *E*_ref_(ν) are the amplitude spectra of THz pulse transmitted through the sample and the reference, respectively. c, $\tilde{n}$, and *d* are the light velocity, the complex refractive index of a sample, and the thickness of a sample, respectively.

### 3.1. Temperature Dependence of Complex Dielectric Constant

The complex refractive index $\tilde{n}$(ν) = *n*(ν) + i*κ*(ν) is determined by the amplitude and phase of the terahertz transmission using Equation (1), where *n*(ν) and κ(ν) are the refractive index and the extinction coefficient. The complex dielectric constant *ε*(ν) = *ε*′(ν) + i*ε*″(ν) is equal to the square of the complex refractive index.

(2) $${\tilde{n}}^{2}\left(\nu \right)={\left(n\left(\nu \right)+i\kappa \left(\nu \right)\right)}^{2}=\tilde{\varepsilon }\left(\nu \right)=\varepsilon^{\prime }\left(\nu \right)+i\varepsilon^{\prime \prime }\left(\nu \right).$$

**Figure 4:**
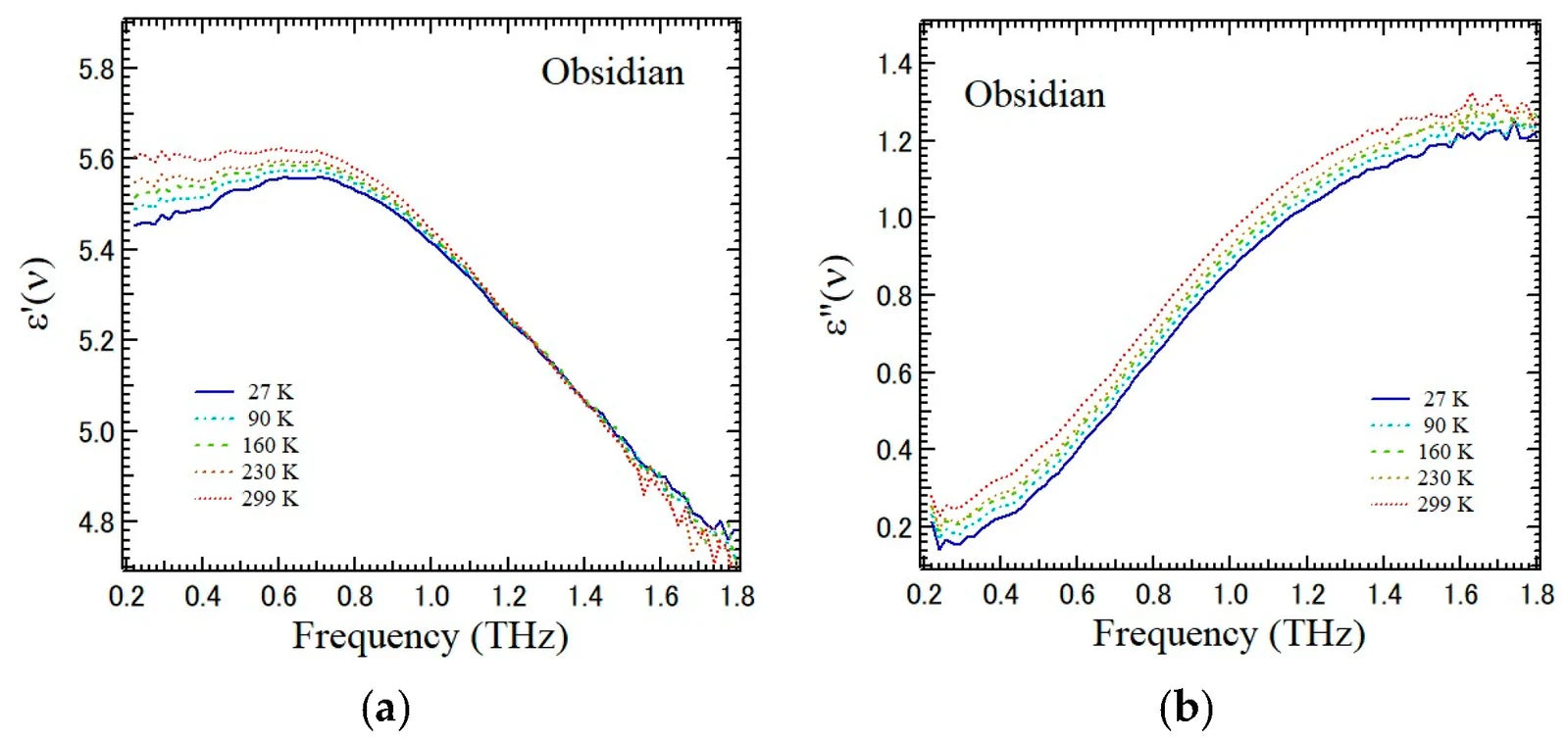
Temperature dependence of (**a**) real and (**b**) imaginary parts of the dielectric constant of obsidian between 27 and 299 K.

The temperature dependences of the real and imaginary parts of the dielectric constant of obsidian were determined from 299 K down to 27 K, as shown in Figure 4a and Figure 4b, respectively.

**Figure 5:**
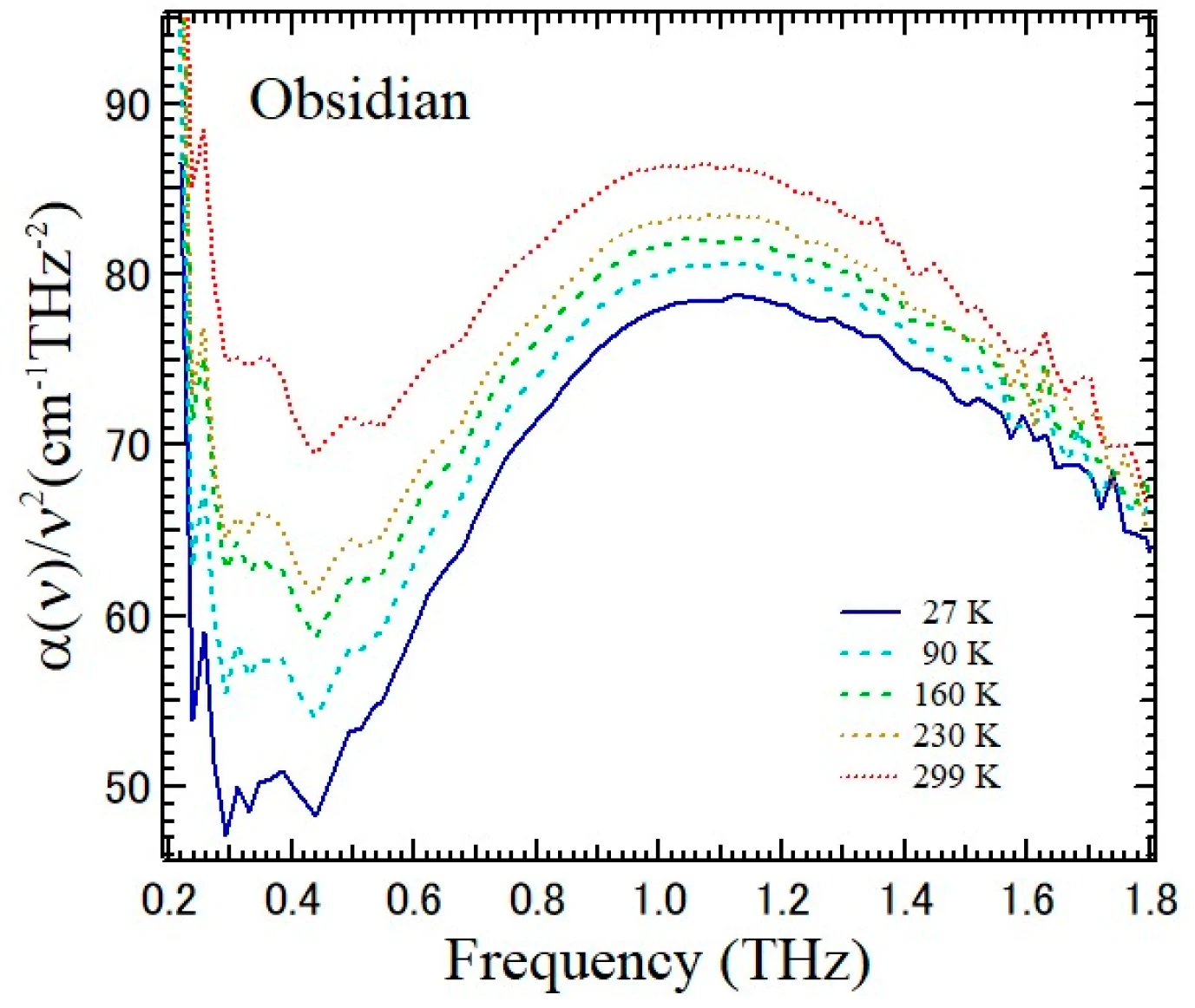
Temperature dependence of boson peak spectra, α(ν)/ν^2^.

The temperature dependence of the real part at 1.2 THz is nearly constant, while that at 0.3 THz decreases by about 2%. Such a decrease indicates the suppression of fast relaxation processes at low temperatures. This frequency range is dominated by a universal feature of glasses, so-called BP, originating from an excess in the VDoS [1,2]. The imaginary part of the dielectric constant is related to VDoS, g(ν), through IR coupling constant C_IR_(ν). A BP spectrum [4] is expressed by

(3) $$\frac{2\pi \cdot \varepsilon^{\prime \prime }(\nu )}{c\cdot n(\nu )\cdot \nu }=\frac{\alpha \left(\nu \right)}{\nu^{2}}=C_{IR}\left(\nu \right)\cdot \frac{g\left(\nu \right)}{\nu^{2}}$$

where C_IR_(ν), α(ν), and g(ν) are the IR coupling constant, absorption constant, and VDoS, respectively. In many kinds of glasses, BPs were observed as asymmetric broad peaks in the sub-THz spectra of *α*(ν)/ν^2^ or ε″(ν)/(*n*(ν)·ν) by THz-TDS [4]. The temperature dependence of α(ν)/ν^2^ of obsidian determined by the complex dielectric constant is plotted to observe a BP, as shown in Figure 5. The BP frequency decreases by about 5% from 1.12 THz at 27 K to 1.07 THz at 299 K. The temperature dependence of the BP frequency was studied in propylene glycol, which was two times larger than that of the longitudinal sound velocity [23]. This variation in obsidian is not significant within experimental uncertainty.

The BP peak frequency of obsidian is comparable with the BP frequency of 1.2 THz of pure silica glass at room temperature [1]. It was reported that the BP frequency of silica glass decreases as the hydro-oxide content increases in the far-IR spectroscopy [24]. The BP frequency of glass A with a very low content of 1 ppm OH was 1.2 THz (41 cm^−1^), while that of the silica glass B with a very high content of 1200 ppm was 1.08 THz (36 cm^−1^). This difference indicates that the content of OH in silica glass increases the overall FIR absorption and decreases the BP frequency from 41 cm^−1^ (glass A) to 36 cm^−1^ (glass B). This effect is consistent with the decrease in the BP frequency in obsidian. However, the OH content of the present obsidian sample was not measured, and its possible contribution to the BP frequency cannot be evaluated in the present study.

The BP frequency also changes by the existence of other metals that modify or break a network of SiO_4_ tetrahedra. The structure of alkali silicate glasses consists of a network of SiO_4_ tetrahedra linked by bridging oxygen atoms (BO). In alkali silicate glasses, the addition of network-modifying cations breaks up the network by generating non-bridging oxygen atoms (NBO) [25]. Such a break of the network may cause an increase in the BP frequency. The BP frequency of SiO_2_-Al_2_O_3_ glasses also increases with the Al_2_O_3_ content [26]. The origin of the BP in binary silicate glasses is attributed to the sum of two independent contributions. One is the rotational or librational motions of interconnected tetrahedral SiO_4_ units, and another is the localized vibrations of the network modifier cations within oxygen cages [27]. We interpret the BP in obsidian as arising from the combined contributions of librations of interconnected SiO_4_ tetrahedra and localized vibrations associated with network-modifying cations within oxygen cages. The mixed alkali and alkali earth effects of the nonlinear additive trend have been studied in borate and silicate glasses in which more than two kinds of metal ions are doped [28]. In obsidian, many kinds of metal ions are included, and the mixed alkali and alkaline earth effects may also induce different types of changes in the BP frequency.

In silica glass, the relation between the BP frequency and the FSDP was well discussed on the basis of the intermediate-range order and coherence length determined by the FSDP. However, to discuss such a relation on obsidian, an accurate measurement of the FSDP by X-ray or neutron diffraction method is required.

### 3.2. Light–Vibration Coupling Constant and Atomic Charge Distribution

The VDoS and the fluctuating atomic charges in a sample play dominant roles as a universal feature of a BP in disordered materials. The contribution from atomic charges is split into two parts. One is an uncorrelated random component due to structural disorder. Another is a correlated component of charge fluctuations due to variations in the local structure, which obeys local charge neutrality within the structural units [29]. To analyze the THz BP peak in THz spectra, the IR light–vibration coupling coefficient, C_IR_(ν), is introduced in Equation (3). A universal model of C_IR_(ν) for terahertz absorption in disordered materials was proposed by Taraskin et al. using the linear response theory with the harmonic approximation for atomic vibrations. C_IR_(ν) depends on the vibrational normal modes and the distribution of atomic charges. For disordered materials, C_IR_(ν) exhibits a universal frequency dependence of the following form in the far IR regime:

(4) $$C_{\mathrm{IR}}(\nu )\;≅\;A+B\nu^{2}$$

where *A* and *B* are material-dependent constants. This law is applicable in the low-frequency part of the far IR domain, approximately up to frequencies on the order of the Ioffe–Regel crossover frequency, ν_IR_. The Ioffe–Regel frequency represents a crossover from well-propagating plane-wave-like excitations to strongly damped excitations due to disorder-induced scattering, rather than a sharp cutoff frequency. The present arguments are based on an analysis of the following general expression for the coefficient of absorption of far-IR photons by harmonic atomic vibrations derived by the rigid-ion model [30]:

(5) $$C_{\mathrm{IR}}(\nu )\;≅C_{0}{\left|\sum_{i}\frac{q_{i}}{\sqrt{m_{i}}}e_{i}|\left(\nu \right)\right|}^{2}$$

where *C*_0_ = 2*π*^2^*n*/*c*$\varepsilon_{\infty }^{1/2}$, *m*_i_ represents the atomic masses, *q*_i_ fixed atomic charges, *e*_i_(ν) the component of the eigenvector of frequency ν corresponding to atom *i*, $\varepsilon_{\infty }$ stands for the high-frequency dielectric constant, and *n* is the atomic concentration.

In the theory of Taraskin et al., *q_i_* is divided into two parts, *q_i_ = q*_1_*_i_* *+ q*_2_*_i_*: the uncorrelated randomly fluctuating charge, *q*_1i_, and the correlated charge that satisfies local charge neutrality, *q*_2i_. It holds that *<q*_1_*_i_q*_1_*_j_> = <q*_1_*_i_> <q*_1_*_j_> = σ*_1_^2^*δ_ij_.* The charges *q*_2i_ satisfy local charge neutrality and can be imagined as resulting from charge transfers between nearest-neighbor atoms, i.e., $q_{2i}=\sum_{i\neq j}∆q_{ji}$, where j runs over all nearest neighbors of atom *i*, and ∆*q*_ji_ = ∆*q*_ij_ is the charge transfer from originally neutral atoms *j* to *i*.

(6) $$\left\langle C_{IR}(\nu )\right\rangle =\frac{2\pi n}{c\sqrt{\bar{m}\varepsilon \infty }}\left\langle N^{-1}{\left|\sum_{i}q_{1}ie^{ik\cdot Ri}|+|\sum_{i}q_{2}ie^{ik\cdot Ri}\right|}^{2}\right\rangle$$

Since there is no correlation between *q*_1_ and *q*_2_,

(7) $$\left\langle C_{IR}(\nu )\right\rangle ≅A+B\nu^{2},\;\mathrm{where}\;A=\frac{2\pi^{2}n}{\bar{m}c\sqrt{\varepsilon_{\infty }}}N^{-1}\langle {\left|\sum_{i}{q_{1i}e}^{ik\cdot R_{i}}\right|}^{2}\rangle =\frac{2\pi^{2}n}{\bar{m}c\sqrt{\varepsilon_{\infty }}}\langle {q_{1i}}^{2}\rangle$$

The frequency-independent part in Equation (7) originates from uncorrelated charge fluctuations, and it holds that

(8) $$\sigma_{1}=\sqrt{\langle {q_{1i}}^{2}\rangle }=\sqrt{\frac{10^{12}\times {\;A}_{\nu }^{fit}\bar{m}c\sqrt{\varepsilon_{\infty }}}{\pi n}}[statC]$$

The quadratic frequency dependence in Equation (7) originates from correlated charge fluctuations that satisfied the local charge neutrality.

In silica glass, $\sigma_{1}$= 0.06*e* was reported [29]. It was also reported that the value of $\sigma_{1}$ increases with the addition of sodium in *x*Na_2_O(1−*x*)SiO_2_ glass, and $\sigma_{1}$ ≈ 0.40e at x = 0.2 [31]. The addition of Na inclusions causes a nonlocal redistribution of all the atomic charges, and a weakening of Si-O bonds occurs. Therefore, long-range uncorrelated charge fluctuations are expected to be enhanced with Na contents. The correlated charge fluctuations of sodosilicate glass were discussed by the observed value of B in Equation (7); however, the significant change was not observed with the sodium concentration. It was suggested that the correlated charge fluctuations are not affected by the sodium inclusions [31].

**Figure 6:**
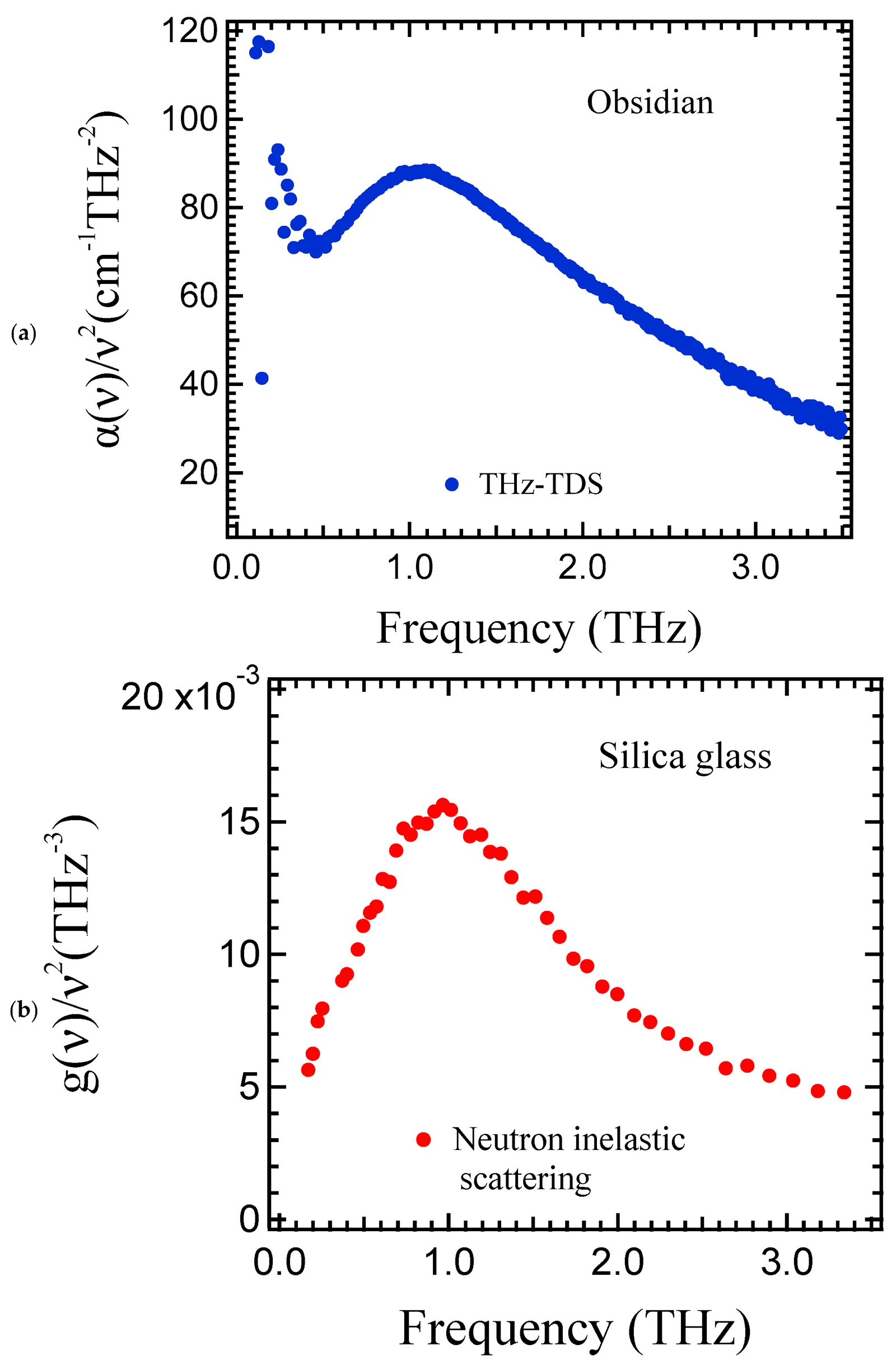
(**a**) Boson peak spectrum of obsidian glass at room temperature. (**b**) VDoS of silica glass observed by neutron inelastic scattering [32].

**Figure 7:**
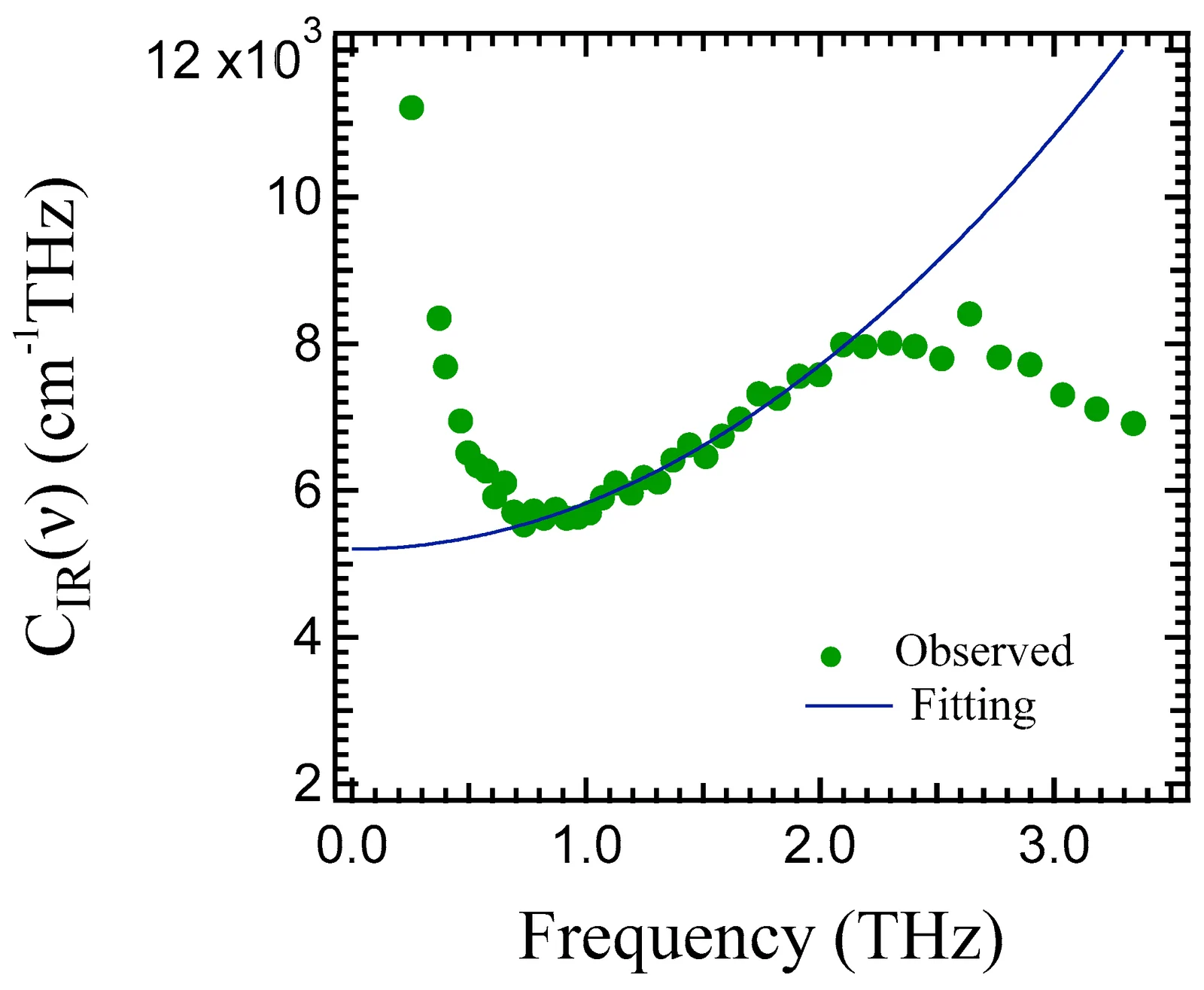
IR coupling constant observed by THz-TDS. Solid line indicates the fitting curve by the Equation (7), where *A* = 5199 ± 47, and *B* = 627 ± 22.

For the estimation of the coupling constant, VDoS of silica glass measured by neutron inelastic scattering in Figure 6b was used to calculate C_IR_(ν) of obsidian, because the chemical composition of obsidian is close to silica glass. The fitting curve for C_IR_(ν) by Equation (7) using the THz spectrum of Figure 6a is shown in Figure 7. The value of *A =* 5199 ± 47 of obsidian was determined. In Equation (7), the value of the atomic concentration n ≈ 6.55452 × 1022 [atom/cm^3^] was calculated using the composition of obsidian in Table 1. The value of the high-frequency dielectric constant ε_∞_ = 4.25 was determined by the real part of the observed dielectric constant in Figure 4a. Using these observed values of obsidian, $\langle {q_{1i}}^{2}\rangle \approx 5.35820\times 10^{-22}[statC^{2}]$, and $\sigma_{1}=\sqrt{\langle {q_{1i}}^{2}\rangle }=0.481e,$ where 1*e* = 1.602176634 × 10^−19^ C, were obtained by Equation (8). This value of obsidian is comparable with that of sodosilicate glass at *x* = 0.2. The content of alkali and alkali earth elements is about 10% in obsidian. The content of 13% aluminum may also cause a nonlocal redistribution of all the atomic charges, which induces a weakening of Si-O bonds.

## 4. Conclusions

A universal excitation called the boson peak has been studied in glassy and amorphous materials in the far-IR range. The broadband terahertz time-domain spectroscopy (THz-TDS) enables the time-of-flight (TOF) detection of not only phonons but also boson peaks. Obsidian is a typical natural inorganic glass, and it is produced by the rapid cooling of volcanic lava through the glass transition temperature and freezes, not permitting sufficient time for crystal growth. The broadband frequency dependence of complex dielectric constants was measured by TOF using THz-TDS. The boson peak at 1.07 THz was observed at room temperature, which is lower than the 1.2 THz of silica glass. The lower BP frequency of obsidian may be attributed to the modulation of the SiO_4_ network by metal ions. The uncorrelated charge fluctuations, $\sigma_{1}=\sqrt{\langle {q_{1i}}^{2}\rangle }=0.481e$ were obtained for obsidian by the analysis of C_IR_ by Taraskin’s model. The value of obsidian is much higher than $\sigma_{1}$ = 0.06*e* of silica glass. However, this value of obsidian is comparable with $\sigma_{1}\approx 0.44e$ of sodosilicate glass, *x*Na_2_O(1-*x*)SiO_2_ at *x* = 0.2 [31]. As a common feature in silica-based glasses, the content of metal elements may lead to a nonlocal redistribution of all the atomic charges, which causes a weakening of Si-O bonds, and the uncorrelated charge fluctuations can be enhanced.

## Figures and Tables

**Table 1:** Composition of obsidian (wt%) measured by X-ray fluorescence analysis.

Element	SiO_2_	Al_2_O_3_	CaO	Na_2_O	K_2_O	Fe_2_O_3_	MgO	TiO_2_	MnO
Content (wt%)	76.49	13.28	0.77	3.98	4.69	0.75	0.06	0.1	0.05

## Data Availability

The original contributions presented in this study are included in the article. Further inquiries can be directed to the corresponding author.

## Abbreviations

The following abbreviations are used in this manuscript:

TOF	Time-of-flight
THz-TDS	Terahertz transmission time-domain spectroscopy
BP	Boson peak
VDoS	Vibrational density of states
FSDP	First sharp diffraction peak
HWHM	Half-maximum half-width
XRF	X-Ray fluorescence analysis
BO	Bridging oxygen atoms
NBO	Non-bridging oxygen atoms
IR	Infrared
